# Oestrogen receptor pathway activity is associated with outcome in endometrial cancer

**DOI:** 10.1038/s41416-020-0925-4

**Published:** 2020-06-08

**Authors:** Willem Jan van Weelden, Louis J. M. van der Putten, Márcia A. Inda, Anne van Brussel, Marc P. L. M. Snijders, Lisanne M. M. Schriever, Johan Bulten, Leon F. A. G. Massuger, Anja van de Stolpe, Johanna M. A. Pijnenborg

**Affiliations:** 1 Department of Obstetrics and Gynaecology, Radboud Institute for Health Science, Radboud university medical center, Nijmegen, the Netherlands; 2 Philips Research, Eindhoven, the Netherlands; 3 Department of Obstetrics and Gynaecology, Canisius-Wilhelmina Hospital, Nijmegen, the Netherlands; 4 Department of Pathology, Radboud university medical center, Nijmegen, the Netherlands

**Keywords:** Endometrial cancer, Cancer microenvironment, Prognostic markers

## Abstract

**Background:**

Oestrogen receptor (ER) expression is a prognostic biomarker in endometrial cancer (EC). However, expression does not provide information about the functional activity of the ER pathway. We evaluated a model to quantify ER pathway activity in EC, and determined the prognostic relevance of ER pathway activity.

**Methods:**

ER pathway activity was measured in two publicly available datasets with endometrial and EC tissue, and one clinical cohort with 107 samples from proliferative and hyperplastic endometrium and endometrioid-type EC (EEC) and uterine serous cancer (USC). ER pathway activity scores were inferred from ER target gene mRNA levels from Affymetrix microarray data (public datasets), or measured by qPCR on formalin-fixed paraffin-embedded samples (clinical cohort) and related to ER expression and outcome.

**Results:**

ER pathway activity scores differed significantly throughout the menstrual cycle supporting the validity of the pathway test. The highest ER pathway scores were found in proliferative and hyperplastic endometrium and stage I EEC, whereas stage II–IV EEC and USCs had significantly lower levels. Low ER pathway activity was associated with recurrent disease, and added prognostic value in patients with low ER expression.

**Conclusion:**

The ER pathway test reflects activity of the ER pathway, and may improve prediction of outcome in EC patients.

## Background

Most patients with endometrial cancer (EC) present with early-stage disease and have favourable outcomes.^1,2^ However, 20% of patients suffer from recurrent disease and have poor outcomes.^3–5^ Approximately half of recurrences occur in patients presumed to have low-risk EC.^6,7^ Therefore, improved identification of patients at risk for recurrence is crucial to prevent over- and undertreatment.

Expression levels of oestrogen receptor (ER) and progesterone receptor (PR), as determined by immunohistochemical (IHC) staining, are established prognostic markers for EC: loss of these receptors is associated with increased risk of lymph node metastasis and recurrence.^5,8^ ER and PR belong to the steroid receptor family and act as ligand-activated transcription factors for oestrogen and progesterone, respectively. Oestrogen and ER play pivotal roles in development of endometrioid-type EC (EEC) as unopposed exposure of the endometrium to oestrogen can lead to endometrial hyperplasia and, subsequently, to EEC.^3^ In contrast to EEC, carcinogenesis of uterine serous cancer (USC) and other types of non-endometrioid-type EC is assumed to be independent of oestrogen, and therefore ER IHC is often absent in USC.^3^

Although recognised as prognostic markers, ER and PR IHC expression alone is not sufficient to identify adverse outcome in all patients.^9–11^ In addition, IHC expression does not provide information about the functional activity of the ER signalling pathway and, hence, does not reflect hormone-driven tumour growth. A novel approach to measure ER signalling pathway activity uses a knowledge-based Bayesian computational model that infers functional activity of the pathway from the expression of mRNAs encoding ER target genes.^12^ Previous studies in breast cancer show that ER pathway activity scores have prognostic value and predict response to endocrine therapy.^12,13^ To date, however, no study has applied the ER pathway test to EC. We hypothesised that the ER pathway test provides insight into the development and progression of EC, and improves the prognostic value of ER IHC expression. Therefore, we validated an Affymetrix and qPCR-based version of the model for ER pathway activity in different types of normal, proliferative and premalignant (hyperplastic) endometrial tissue. Furthermore, we investigated EC tissue to determine whether ER pathway activity adds prognostic value to ER IHC expression.

## Methods

### Publicly available datasets

#### Endometrial tissue Affymetrix dataset

This dataset contains Affymetrix U133 Plus 2.0 gene expression data from endometrium samples from four publicly available datasets in the Gene Expression Omnibus repository. Dataset GSE29981 was deposited in 2011 and contains 20 endometrium samples taken at different phases of the menstrual cycle with annotation of the day in the menstrual cycle and the serum progesterone level.^14^ Dataset GSE51981, from 2013, has 69 normal endometrium samples annotated for the menstrual cycle phase based on endometrial histology, reviewed by two pathologists and confirmed by serum oestradiol and progesterone levels.^15^ Dataset GSE6364, from 2006, includes 16 samples from patients with normal endometrium. Dating of the biopsies was based on the reported day of the menstrual cycle, and confirmed by up to four independent histopathologists blinded for the timing of the biopsy.^16^ Finally, dataset GSE12446, from 2008, contains data from 31 endometrium samples of postmenopausal women treated with oestradiol, medroxyprogesterone acetate or tibolone for 21 days before undergoing vaginal hysterectomy for uterine prolapse.^17^

#### EC Affymetrix dataset

The EC Affymetrix dataset comprised Affymetrix U133 Plus 2.0 gene expression data from GEO datasets GSE56026 and GSE2109.^18,19^ Dataset GSE56026, from 2014, includes gene expression microarray data for 63 patients with EEC or USC.^18^ GSE2109 is the International Genomics Consortium database containing gene expression data from more than 2000 cancer patients, including 200 patients with EC.^19^ The gene expression data for this dataset were made public in 2004.

### Nijmegen clinical cohort

In this cohort, ER and PR IHC expression and the qPCR-based version of the ER pathway test was performed in proliferative endometrium samples, hyperplastic endometrial samples and samples of patients who were surgically treated for endometrial carcinoma at the Radboud university medical center or Canisius-Wilhelmina Hospital in Nijmegen between 1999 and 2009.^20,21^ Proliferative endometrium tissue samples were taken from premenopausal women who were in the proliferative phase of the menstrual cycle. Endometrial hyperplasia samples originated from postmenopausal women who underwent a hysterectomy at the Radboud university medical center between 2002 and 2012, and had histologically proven endometrial hyperplasia without malignancy. Pathological review of all cases was performed by an expert gynaecological pathologist (JB).

#### Immunohistochemical staining and scoring

In the Nijmegen clinical cohort, ER and PR IHC expression was analysed on two 4-µm tumour-containing sections from formalin-fixed paraffin-embedded (FFPE) tumour blocks. The IHC expression was performed as described before.^8^ For a complete description, see the Supplementary information. The percentage of tumour cells expressing nuclear ERα and PR was independently evaluated by two researchers with experience in evaluation of IHC slides (LP and LS).^8^ In the case of major disagreement, the final score was settled by an expert gynaecological pathologist (JB). All reviewers were blinded for clinical and pathological data.

#### RNA isolation

In samples from the Nijmegen clinical cohort, marked tissue of interest was microdissected from two consecutive 10-µm FFPE sections. RNA was extracted using the miRNeasy FFPE Kit (Qiagen, Hilden, Germany) according to the instructions of the manufacturer, and as described in full in the supplementary information.

### ER pathway analysis

Development of the ER pathway test has been described in detail.^12^ The model uses mRNA expression levels of ER target genes measured in a tissue sample to infer the odds in favour of a transcriptionally active ER transcription factor and, as a consequence, odds in favour of an active ER pathway. A Bayesian network representing the ER pathway transcriptional programme describes how target gene regulation depends on ER transcription complex activity and how expression-level intensities in turn depend on regulation of the respective target genes (Supplementary Fig. 1). The network consists of three type of nodes: (i) ER transcription complex activation node, (ii) target gene regulation node, with states “down” and “up” and (iii) expression-intensity nodes, with states “low” and “high, each corresponding to an ER target gene. For the public datasets, ER pathway activity was measured using an optimised version of the Affymetrix HGU133 Plus 2.0 (AffyPlus2.0) microarray platform model (affyP2 ER-A1.0a model).^22–24^ Prior to data analysis, an extensive quality control procedure was performed on the Affymetrix data, as described in detail before.^25^ Since Affymetrix U133 Plus 2.0 microarray data cannot be extracted from FFPE material in the Nijmegen clinical cohort, the Affymetrix-based model was adapted for use on qPCR-based mRNA measurements generated by Philips (qPCR ER-E2015 model, Philips Electronics, Eindhoven, The Netherlands, www.philips.com/oncosignal). The procedure for this adaption has been described in detail by Inda et al.^13^ In brief, based on analysis of multiple Affymetrix expression microarray datasets using the originally described ER pathway model, the best-performing set of target genes was selected.^12^ Subsequently, for each target gene, a qPCR assay was developed and validated, while a set of qPCR tests performed on reference genes were used for normalisation of PCR results. Using the multiple target gene qPCR set, the ER pathway model was calibrated on the MCF7 breast cancer cell line, either deprived from or stimulated with oestradiol to generate “ground truth” samples. This qPCR-based ER pathway test, consisting of multiple independent qPCR measurements of target and reference genes, and a calibrated Bayesian computational model for measurement interpretation, was validated on independent samples with a known ER pathway activity (e.g., cell lines stimulated with oestradiol, and/or inhibited with fulvestrant). The qPCR-based model has shown good agreement with the Affymetrix-based model.^13^ To further facilitate the comparison between results obtained on different measurement platforms, odds in favour of an active ER pathway were transformed into a base 2 logarithmic scale and normalised against a pathway activity score, such that the resultant values lay between 0 and 100, where 0 corresponds to the lowest odds for pathway activity, and 100 corresponds to the maximum odds for pathway activity that the model can infer. All samples were analysed in a blinded manner.

### Statistical data analysis

The ER pathway test was validated in the endometrial tissue Affymetrix dataset by analysis of ER pathway activity in different phases of the menstrual cycle. Differences in ER pathway activity among proliferative and secretory, and inactive and oestrogen-stimulated endometrium were analysed using one-way ANOVA with Tukey’s HSD post hoc test. The differences in clinicopathological characteristics in the EC Affymetrix dataset and Nijmegen clinical cohort were analysed using the Mann–Whitney U test (continuous variables) and the χ^2^ test (categorical data). One-way ANOVA with Tukey’s HSD post hoc test was used to analyse differences in ER pathway activity score in proliferative and hyperplastic endometrium, as well as in low and high grade, stage I and stage II–IV EEC and NEEC histology. The relation between ER pathway activity score and ER and PR IHC expression was explored in three ER and PR IHC expression groups (0–10%, 11–50% and 51–100%). The association between ER pathway activity and disease-free survival (DFS) was analysed with univariate Cox regression analysis among all EC-patients in the Nijmegen clinical cohort, and for  ER/PR IHC subgroups specifically. Kaplan–Meier analyses with log-rank tests were performed to show DFS and DSS relative to ER IHC expression and ER pathway activity. Patients with residual disease were excluded from analyses involving DFS. A cut-off of 10% for ER IHC expression was used to differentiate between low and high ER expression for this analysis as this cut-off value is assumed to have the best association with outcome.^5^ For ER pathway activity, a cut-off is yet to be determined in EC. Therefore, we grouped patients according to ER pathway activity to determine the optimal relation with outcome. The association between DFS and DSS and prognostic factors, including ER pathway activity, ER IHC expression and other prognostic factors, was analysed by univariate Cox regression analysis. Factors with a significant association in univariate analysis were included in multivariate regression analysis. Differences were considered significant at a two-sided *P* value ≤ 0.05. SPSS version 22 (SPSS IBM, New York, NY, USA) and GraphPad Prism 5.03 were used to perform the statistical analyses.

### Ethical approval

This study was performed in accordance with the Declaration of Helsinki, and was approved by the Institutional Review Board at the Radboud university medical center (2016–2285). The need to obtain consent was waived based on the code of conduct for responsible use of human tissue in medical research.^26^

## Results

### Public datasets

#### Endometrial tissue Affymetrix dataset

**Fig. 1:**
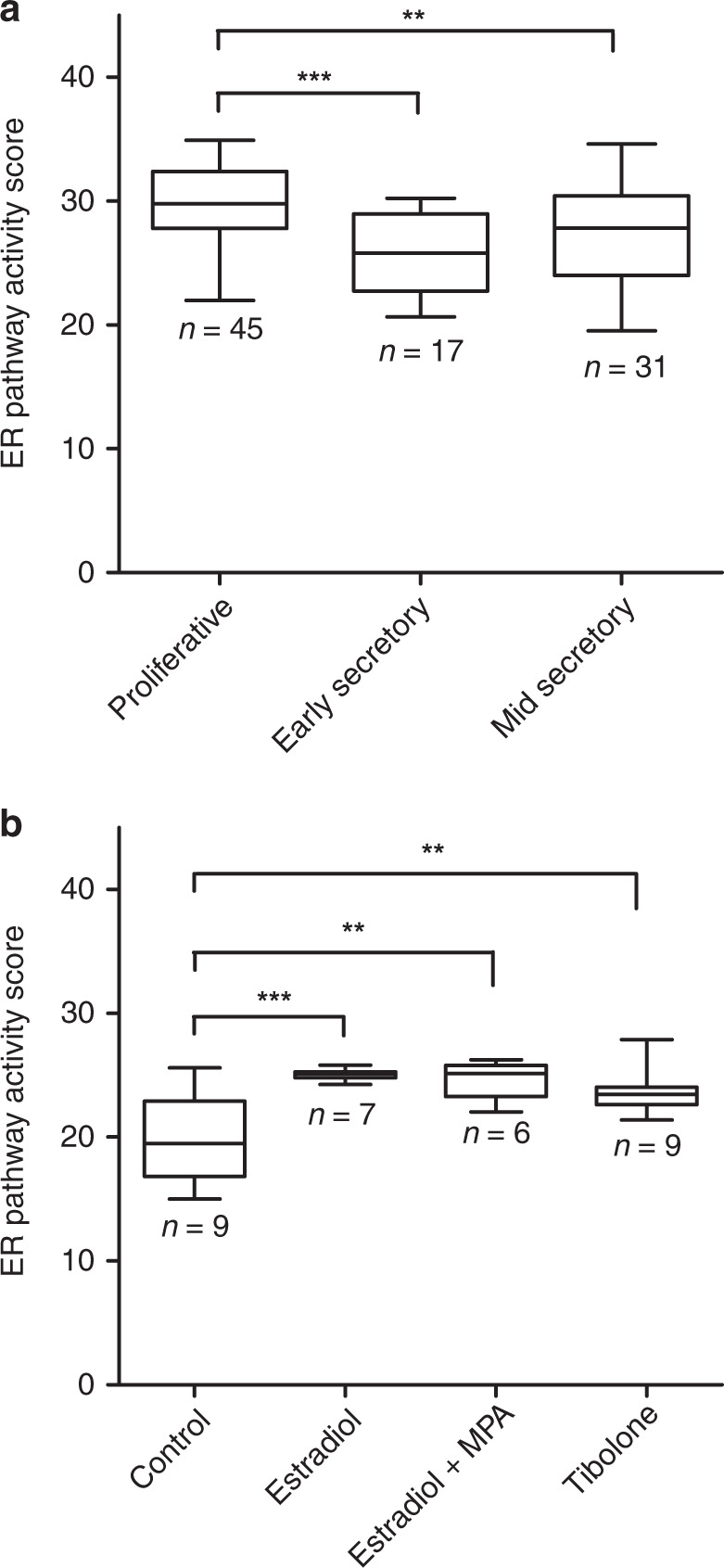
Validation of the oestrogen receptor pathway test in normal endometrium from the endometrial tissue Affymetrix dataset. **a** Differences in ER pathway activity scores at the proliferative, early secretory and mid-secretory phase of the menstrual cycle, as measured in datasets GSE29981, GSE51981 and GSE6364. **b** Differences in ER pathway activity scores in postmenopausal women with inactive endometrium and endometrium treated with oestradiol, medroxyprogesterone acetate (MPA) or tibolone. Data are from dataset GSE12446. **P ≤ 0.01; ***P ≤ 0.001.

A total of 105 cyclic endometrium samples originating from datasets GSE29981, GSE51891 and GSE6364 were included. Fourteen samples from GSE29981 were excluded: twelve because they failed the quality control checks and two because classification of the menstrual phase was unclear, leaving a total of 93 high-quality samples. The ER pathway activity was significantly higher in the proliferative phase compared with the early and mid-secretory phases of the menstrual cycle (Fig. 1a). This is in line with known oestrogen levels during the menstrual cycle. A total of 31 high-quality endometrial samples of postmenopausal women treated with oestradiol and medroxyprogesterone acetate from dataset GSE12446 were included. ER pathway activity scores were the lowest in inactive endometrium samples from postmenopausal women. Administration of oestradiol, medroxyprogesterone acetate (MPA) or tibolone resulted in a significant increase in ER pathway activity (Fig. 1b).

#### EC Affymetrix dataset

**Fig. 2:**
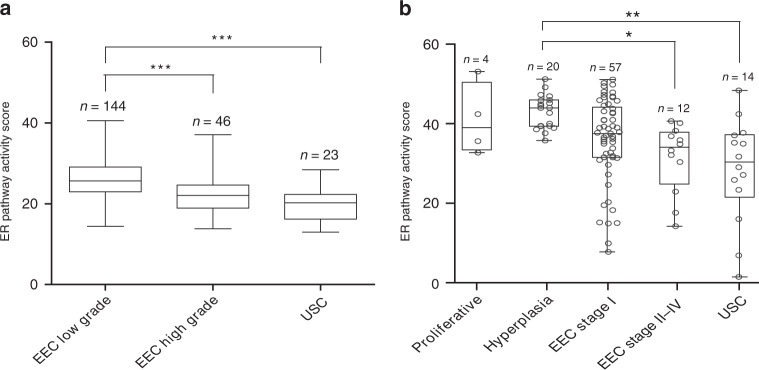
ER pathway activity in proliferative endometrium, premalignant (hyperplastic) endometrium and endometrial cancer in the EC Affymetrix dataset and Nijmegen clinical cohort. **a** ER pathway activity scores according to endometrial cancer grade and histology in the EC Affymetrix dataset (datasets GSE56026 and GSE2109). **b** ER pathway activity scores in proliferative endometrium (benign), endometrial hyperplasia (premalignant) and various types of endometrial cancer in the clinical cohort. EEC endometrioid-type endometrial cancer, USC uterine serous cancer. Proliferative: proliferative endometrium; hyperplasia: endometrial hyperplasia. Dots represent samples from individual patients. ***P ≤ 0.001; **P ≤ 0.01; *P < 0.05.

The clinicopathological characteristics of the EC Affymetrix dataset are shown in the appendix (Supplementary Table 1). ER pathway activity according to tumour grade and histology is shown in Fig. 2a. Patients with low-grade EEC had significantly higher ER pathway activity than patients with high-grade EEC or those with USC.

#### Nijmegen clinical cohort

In total, 83 EC patients with available FFPE samples were included in this cohort: 57 patients with stage I EEC, 12 with stage II–IV EEC and 14 with USC. Clinicopathological characteristics of the EC patients are provided in Table 1. Included EC patients had a follow-up of at least 36 months, unless patients died or suffered from  a recurrence within this period. Furthermore, we included endometrial samples from four premenopausal women with normal proliferative endometrium, and 20 postmenopausal women with endometrial hyperplasia without malignant disease in this cohort.

**Table 1:** Clinicopathological characteristics of the endometrial cancer patients included in the study.

	EEC stage I	EEC stage II–IV	USC
Number of patients	57	12	14
Age^a^ (range)	62 (45–80)	64 (53–82)	71 (58–82)
Follow-up^a^ (range)	57 months (6–123)	45 months (3–71)	28 months (6–74)
*Treatment*			
Lymphadenectomy	8 (14%)	7 (58%)	12 (86%)
Radiotherapy	15 (26%)	10 (83%)	5 (36%)
Chemotherapy	–	3 (25%)	8 (57%)
*FIGO stage*			
I	57 (100%)	–	4 (29%)
II	–	4 (33%)	–
III	–	5 (42%)	3 (21%)
IV	–	3 (25%)	7 (50%)
*Grade*			
1 or 2	51 (89%)	7 (58%)	–
3	6 (11%)	5 (42%)	14 (100%)
*Myometrial invasion*			
<1/2	42 (74%)	3 (25%)	6 (43%)
≥1/2	15 (26%)	9 (75%)	8 (57%)
*LVSI*			
No	46 (81%)	3 (25%)	5 (36%)
Yes	11 (19%)	9 (75%)	8 (57%)
*Recurrence*^b^			
Yes	8 (14%)	6 (55%)	6 (60%)
Distant	5 (9%)	6 (55%)	5 (60%)
No	49 (86%)	5 (46%)	4 (40%)
*EC-related mortality*			
Yes	4 (7%)	5 (42%)	8 (57%)
No	53 (93%)	7 (58%)	6 (43%)

*LVSI* lymphovascular space invasion.

^a^Median value reported.

^b^Analysis among patients without residual disease only.

#### ER pathway activity scores and ER/PR IHC expression

Normal proliferative endometrium samples from premenopausal women had a mean ER pathway activity score of 41 (SD 8); this was not different from endometrial hyperplasia samples from postmenopausal women (mean 43 (SD 4)) or EEC stage I patients (mean 36 (SD 11)) (Fig. 2b). ER pathway activity was significantly higher in the hyperplasia group than in the EEC stage II–IV (mean 31, SD 9) and USC (mean 28, SD 13) groups. The difference between EEC stage I and stage II–IV was not significant (*P* = 0.395).

Analyses stratified by grade and histology showed that low-grade EEC had a significantly higher ER pathway activity (mean score: 37.3, SD 8.8) compared with high-grade EEC (mean score: 23.7, SD 10.8, *P* < 0.001) and USC (mean 28.4, SD 13.1, *P* = 0.009). The difference between high-grade EEC and USC was not significant (*P* = 0.478).

**Fig. 3:**
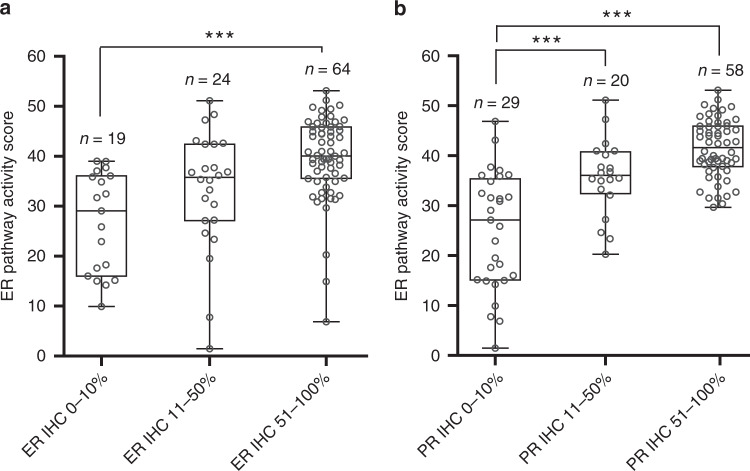
ER pathway activity score according to (**a**) ER IHC expression and (**b**) PR IHC expression in the Nijmegen clinical cohort. Dots represent samples from individual patients. ***P ≤ 0.001.

Figure 3 shows the correlation between ER pathway activity and ER and PR IHC expression. ER pathway activity in the group with low ER IHC expression (0–10%) was significantly lower than in the group with high ER IHC expression (51–100%) (Fig. 3a). ER pathway activity exhibited similar significant correlations with PR IHC expression (Fig. 3b).

#### Clinical outcome

**Fig. 4:**
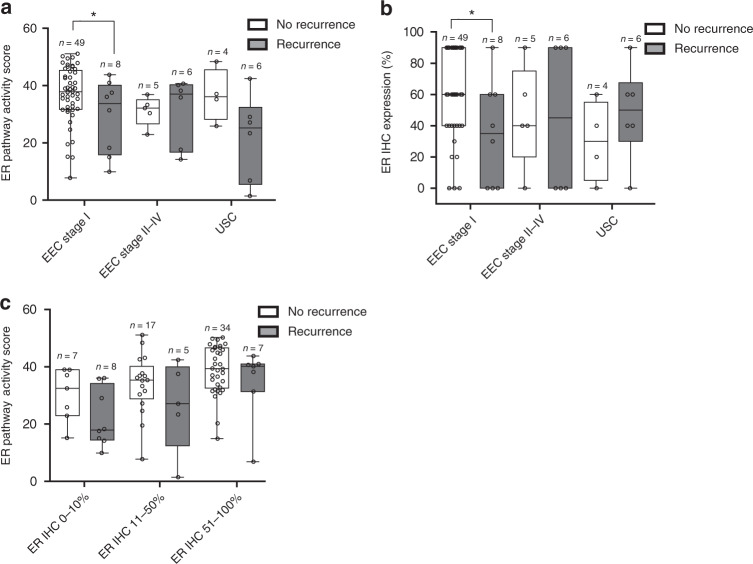
Relationship between ER pathway activity score and ER IHC expression in patients with and without a recurrence in the Nijmegen clinical cohort. **a** ER pathway activity scores in patients with and without a recurrence, according to stage and histology. **b** ER IHC expression in patients with and without a recurrence, according to the stage and histology. **c** ER pathway activity score in patients with and without a recurrence, according to ER IHC expression groups. Dots represent samples from individual patients, five patients with residual disease were excluded from this analysis (USC: *n* = 4, EEC stage II–IV: *n* = 1). EEC endometrioid-type endometrial cancer, USC uterine serous cancer. *P < 0.05.

Overall, EC patients that developed a recurrence had significantly lower ER pathway activity scores than patients without a recurrence: mean score 26 (SD 14) versus 37 (SD 9), respectively (*P* = 0.001). Among patients with EEC stage I, mean ER pathway activity was significantly lower in patients with recurrence than in patients without recurrence (Fig. 4a). There was no significant difference in the ER pathway activity in the EEC stage II–IV and USC groups with and without a recurrence, although the mean ER pathway activity score in USC patients without a recurrence appeared to be higher compared with recurring patients. ER IHC expression of EEC stage I patients with a recurrence was also significantly lower than that in patients without a recurrence (Fig. 4b). When grouped according to ER IHC expression (0–10%, 11–50% and 51–100%), no significant differences in ER pathway activity were found between patients with and without recurrence, although there was a trend towards lower ER pathway activity in patients with a recurrence in ER IHC 0–10% (*P*0.094, Fig. 4c).

**Fig. 5:**
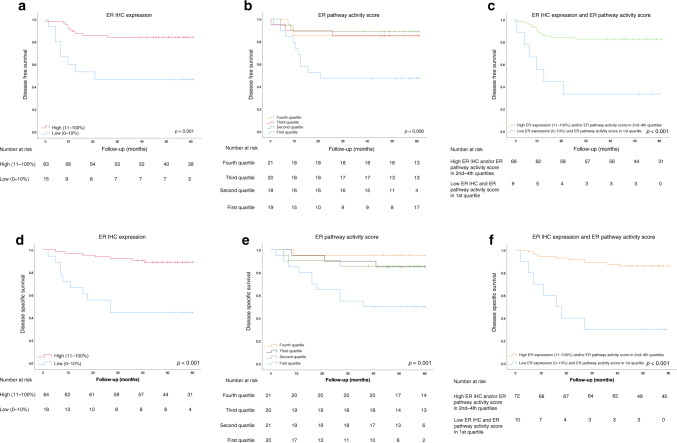
Disease-free and disease-specific survival according to ER IHC expression and ER pathway activity in the Nijmegen clinical cohort. **a** Kaplan–Meier curve for DFS according to ER IHC expression; cut-off, 10%. **b** Kaplan–Meier curve for DFS according to ER pathway activity, divided into quartiles. **c** Kaplan–Meier curve for DFS for patients with combined low ER IHC expression (0–10%) and ER pathway activity in the first quartile, relative to patients with high ER IHC expression (11–100%) or ER pathway activity in the second-to- fourth quartiles. **d** Kaplan–Meier curve for DSS according to the ER IHC expression; cut-off, 10%. (**e**) Kaplan–Meier curve for DSS according to ER pathway activity, divided into quartiles. **f** Kaplan–Meier curve for DSS for patients with combined low ER IHC expression (0–10%) and ER pathway activity in the first quartile, relative to patients with high ER IHC expression (11–100%), and/or ER pathway activity in the second-to-fourth quartiles. Five patients with residual disease were excluded from panels **a**–**c**.

Kaplan–Meier survival analysis revealed a significant association between a low ER IHC expression (0–10%) and reduced DFS and DSS (Fig. 5a, d). Similarly, an ER pathway activity score in the lowest quartile was associated with reduced DFS and DSS (Fig. 5b, e). Patients with both low ER IHC expression and an ER pathway activity score in the lowest quartile had the shortest DFS and DSS (Fig. 5c, f).

Univariate Cox regression analysis revealed that tumour grade, lymphovascular space invasion (LVSI), deep myometrial invasion (deep MI), FIGO stage, ER IHC expression and ER pathway activity were associated with reduced 5-year DFS and DSS. In multivariate analysis, all parameters lost significance, except for FIGO stage for DFS and deep MI for DSS (*P* = 0.022 and *P* = 0.044, respectively) (Supplementary Table 2).

## Discussion

In this study, we show that the ER pathway test correctly identified expected differences in ER pathway activity during the menstrual cycle and in the endometrium that is stimulated with oestradiol or partial oestrogen analogues. This confirmed that the ER pathway test, which was originally developed for use in breast cancer samples, can be used without modification on Affymetrix microarray data from endometrial tissue samples. Because Affymetrix microarray analysis cannot be performed on FFPE material, the ER pathway test had to be adapted to enable qPCR-based measurement on FFPE material. We observed a similar relationship between ER pathway activity and tumour grade and stage in the EC Affymetrix dataset and qPCR-based data (Nijmegen clinical cohort), indicating that the qPCR-adapted ER pathway model adequately reflects differences in ER pathway activity. The positive correlation between ER and PR IHC expression and ER pathway activity in the Nijmegen clinical cohort supported the validity of the qPCR-adapted ER pathway model.^12^

The ER pathway activity scores in postmenopausal patients with endometrial hyperplasia or EEC stage I were similar to activity scores in premenopausal women with proliferative endometrium, indicating that inactive postmenopausal endometrium can be stimulated by oestrogens to premenopausal ER pathway activity levels. This is in line with current theories on the stimulatory effect of oestrogens in the development of endometrial hyperplasia and EEC.^27^ Our study also showed that USC is associated with lower ER pathway activity, although a selection of USCs had an ER activity comparable with that in stage I EECs, indicating biological variation in USCs.^28,29^ Validation in a larger database is necessary to confirm differences in ER pathway  activity in patients with USC. The lower ER pathway activity in patients with EEC stage I that developed a recurrence and advanced-stage EEC suggests a relationship between inactivation of the ER pathway and tumour progression and recurrence. These findings are supported by studies reporting a relationship between loss of ER IHC expression and activation of the TGF-β pathway and other pathways that are involved in the metastatic process.^10,30–32^ Further studies on the interplay between the ER and signal transduction pathways involved in metastasis will provide more insight into the relation between inactivation of the ER pathway and the process of metastasis. Within the group of advanced-stage EC, there were no differences in ER pathway activity, possibly due to the low number of included cases (data not shown).

ER pathway activity was associated with ER and PR IHC expression, as well as with clinical outcome. The variation in ER pathway activity scores observed within the three ER IHC expression groups can be explained by the large range in ER IHC expression within a group (e.g., between 10% and 50% positive cancer nuclei). Further contributing to this variation is the fact that the presence of ER is necessary for ER pathway activity, but not always sufficient, as reported for breast cancer.^12,33^ With the exception of rare ER-activating mutations, locally present oestradiol is required to activate the ER, and availability of such factors depends on menopausal status and local oestrogen production (e.g., from fat cells or local aromatase). Thus, ER pathway activity might be a better reflection of the actual activation of the ER pathway than ER IHC expression. That could explain the added prognostic value of the ER pathway test in the identification of patients with adverse outcome. In the multivariate analysis, no significant association between clinical outcome and either ER IHC expression or ER pathway activity was observed; this could be due to the limited number of patients with advanced EEC and USC included in the study.

Apart from the prognostic value of the ER pathway test, it would be relevant to study the predictive value of the ER pathway test for response to hormonal therapy in EC. Hormonal therapy has a limited role in EC, and is mainly prescribed for fertility preservation and in a palliative setting for patients with advanced or recurrent EC.^34^ To some extent, the response to hormonal therapy can be predicted by ER and PR IHC scores, but further optimisation is necessary.^35,36^ Measurement of ER pathway activity may have additional predictive value in this context.

This is the first study to investigate the functional activity of the ER pathway in EC. One limitation of this study is the small number of samples in the Nijmegen clinical cohort. Another limitation is the lack of comparability between the Affymetrix and qPCR-based models for ER pathway activity. However, we validated both models for application in EC, and acquired similar results with both tests. Thus, further studies can be performed on both fresh frozen and FFPE tumour tissues. Considering that FFPE tumour tissue storage is routine practice in pathological laboratories throughout the world, validation of the qPCR-based model in FFPE will greatly facilitate application of the ER pathway activity test in clinical practice. Other strengths of this study include selection of a representative clinical cohort and validation of the ER pathway test in endometrial tissue. Absolute ER pathway activity scores in EC are lower than those in breast cancer; therefore, definition of an EC-specific range of ER pathway scores could facilitate further clinical use.^13^

In conclusion, we show that high ER pathway activity is associated with the development of EEC, but appears to be less relevant for EEC progression and development of USC. Low ER pathway activity was associated with adverse outcome, and improved the prognostic value of ER IHC expression. Further studies are needed to investigate the role of inactivation of the ER pathway in the process of metastasis, and to confirm the additional prognostic and predictive effect of ER pathway activity.

## Supplementary information

Supplementary figure 1

Supplementary material

## Data Availability

Data from the Nijmegen clinical cohort used during this study can be made available from the corresponding author on reasonable request.
