# Supplementary figures and images for: Oestrogen receptor pathway activity is associated with outcome in endometrial cancer
Source: Br J Cancer. 2020 Jun 8;123(5):785–92. doi: 10.1038/s41416-020-0925-4 (PMC7463017; doi:10.1038/s41416-020-0925-4)

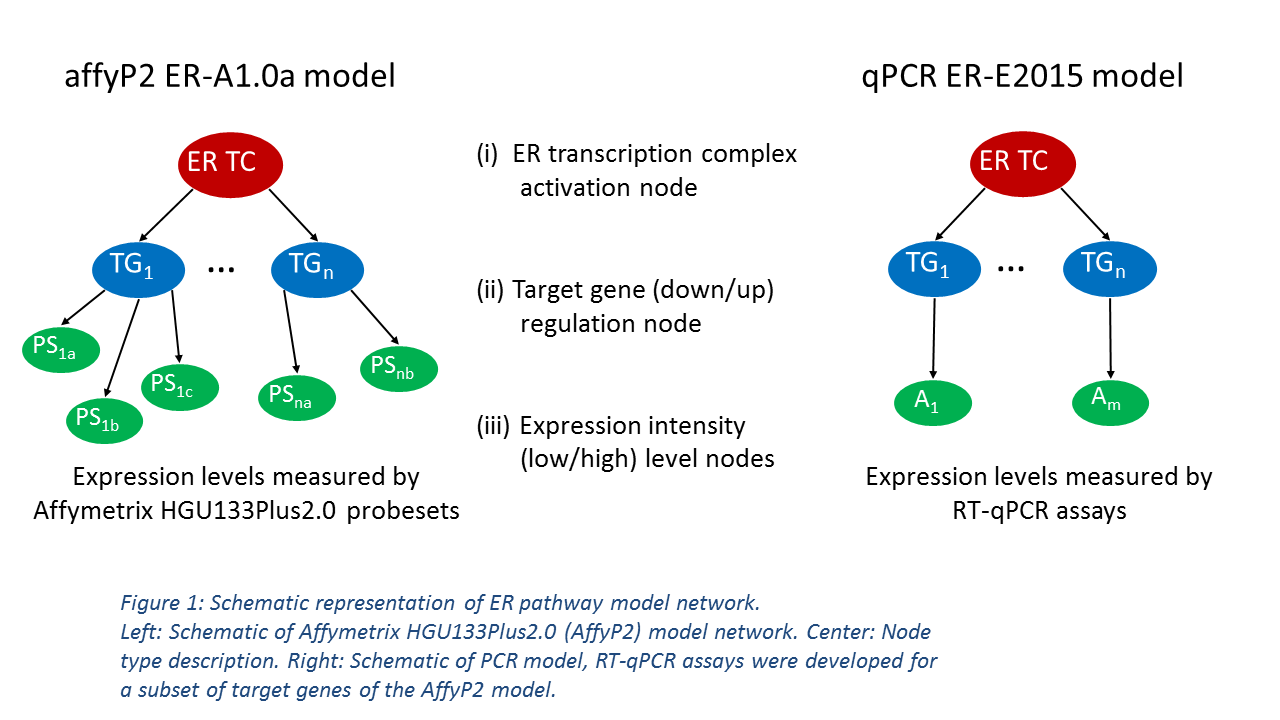

Supplement: Supplementary file 1 — Supplementary figure 1 [file 41416_2020_925_MOESM1_ESM.tif]
