# Supplementary material for: Oestrogen receptor pathway activity is associated with outcome in endometrial cancer
Source: Br J Cancer. 2020 Jun 8;123(5):785–92. doi: 10.1038/s41416-020-0925-4 (PMC7463017; doi:10.1038/s41416-020-0925-4)
Supplement: Supplementary file 2 — Supplementary material [file 41416_2020_925_MOESM2_ESM.docx]

**Supplementary information**

*Complete protocol for IHC expression procedure*

ER and PR IHC expression was analyzed on two 4- µm tumor-containing setion from formalin-fixed paraffin-embedded (FFPE) tumor blocks that were cut and mounted on Superfrost slides. After EDTA antigen retrieval and blocking of endogenous peroxidase with hydrogen peroxide, slides were incubated with either ERα antibody (SP1 RM-9101-S, Thermo Scientific Immunologic, Waltman, MA, USA) diluted 1:40 in normal antibody diluent (Immunologic BV, Duiven, the Netherlands), or PR antibody (PgR636 PR antibody, Dako, Denmark) diluted 1:500 in normal antibody diluent. The slides were subsequently incubated with PowerVision+ Poly-HRP (Leica Microsystems, Buffalo Grove, IL, US) antibodies 1:1 diluted in 1x PBS containing 0.05% Tween 20, and then visualized with PowerVision DAB substrate solution (Leica Biosystems, Buffalo Grove, IL, US). Finally, the slides were counterstained with hematoxylin, dehydrated, and mounted. For internal controls, breast and liver tissue slides with known ERα and PR IHC status were stained as described above.

*Complete protocol for RNA isolation*

In samples from the Radboudumc clinical cohort, marked tissue of interest was microdissected from two consecutive 10-µm FFPE sections. Subsequently, the tissue was transferred into 2-ml microcentrifuge tubes. RNA was extracted using the miRNeasy FFPE Kit (Qiagen, Hilden, Germany) with an optimized protocol. Briefly, First, the tissue was incubated for 15 minutes at 60°C and 15 minutes at 80°C with 240 µl buffer PKD and 50 µl Proteinase K. after which 500 µl buffer RBC was added. The sample was transferred into a QIAmp mini-spin column (Qiagen, Hilden, Germany) and centrifuged for 30 seconds at 10 000 rpm. The flow-through was mixed with 1200 µl 100% ethanol, and 700 µl of the sample was transferred to a new RNeasy MinElute spin column and centrifuged for 15 seconds; the flow-through was discarded. This step was repeated until the entire sample had passed through, after which 500 µl buffer RPE was added, and the sample was centrifuged for 15 seconds at 10 000 rpm with a closed lid. Another 500 µl buffer RPE was added, and the sample was centrifuged for 2 minutes at 10 000 rpm. The spin column was then placed in a 2-ml microcentrifuge tube, centrifuged for 2 minutes with a closed lid and 5 minutes with an open lid, both at 10 000 rpm. After the flow-through was discarded, the spin column was placed in a 1.5-ml microcentrifuge tube, and 30 µl RNase-free water was put directly onto the membrane of the spin column, and centrifuged for 1 minute at full speed with a closed lid. This step was repeated to generate higher yield and total volume. To ensure complete removal of DNA, the eluate was treated for 30 minutes at 37°C with 7.2 µl DNase solution from the Siemens VERSANT Tissue Prep Reagent kit.

| **Supplementary table 1** Clinicopathological findings of patients included in the EC dataset | | | | | | |
| --- | --- | --- | --- | --- | --- | --- |
|  | | |  |  |  |  |
|  | | | **Combined** | **GSE2109** | **GSE56026** | **p^a^** |
| **Number of patients** | | | 263 | 200 | 63 |  |
| **Median age (years)** | | | 65 (range 35–85) | 65 (range 35–85) | NA | 1.000^b^ |
| **Grade**  1 or 2  3  Unknown | | | 148 (56%)  90 (34%)  25 (10%) | 108 (54%)  67 (34%)  25 (13%) | 40 (63%)  23 (37%) | 0.803 |
| **FIGO stage**  I  II–IV  Unknown | | | 128 (49%)  90 (34%)  45 (17%) | 97 (49%)  58 (29%)  45 (23%) | 31 (49%)  32 (51%) | <0.001 |
| **Histology**  Endometrioid  Serous  Unspecified | | | 209 (80%)  23 (9%)  31 (12%) | 158 (79%)  11 (5%)  31 (16%) | 51 (81%)  12 (19%) | <0.001 |
| ^a^ χ^2^ test  ^b^ Mann–Whitney U-test  Data from the Affymetrix databases GSE2109 and GSE56026 | | | | | | |
|  |  |  |  |  |  |  |

**Supplementary table 2**: Univariate and multivariate Cox regression analysis of the association of disease-free survival (DFS) and disease-specific survival (DSS) with prognostic factors including ER IHC expression and ER pathway activity.

| **Disease-free survival** | **Univariate HR (95% CI), *p*-value** | **Multivariate HR (95% CI), *p*-value** |
| --- | --- | --- |
| **Age** | 1.83 (0.60–5.56). p=0.287 |  |
| **Tumor grade** | 10.11 (3.58–28.53), p<0.001 | 2.82 (0.64–12.45), p=0.172 |
| **LVSI** | 4.64 (1.72–12.56), p=0.003 | 1.27 (0.35–4.63), p=0.721 |
| **Deep MI** | 2.72 (1.07–6.90), p=0.035 | 1.37 (0.46–4.04), p=0.573 |
| **FIGO stage** | 11.85 (4.53–30.99), p<0.001 | 4.71 (1.24–17.82), p=0.022 |
| **ER IHC expression** | 4.56 (1.80–11.60), p=0.001 | 2.35 (0.72–7.69), p=0.157 |
| **ER pathway activity** | 4.60 (1.81–11.70), p=0.001 | 0.93 (0.24–3.65), p=0.920 |

| **Disease-specific survival** | **Univariate HR (95% CI), *p*-value** | **Multivariate HR (95% CI), *p*-value** |
| --- | --- | --- |
| **Age** | 2.377 (0.68–8.28), p=0.174 |  |
| **Tumor grade** | 26.202 (5.96–115.17), p<0.001 | 4.35 (0.63–30.05), p=0.136 |
| **LVSI** | 10.875 (3.09–38.22), p<0.001 | 3.69 (0.98–13.94), p=0.054 |
| **Deep MI** | 6.736 (2.19–20.71), p=0.001 | 4.26 (1.04–17.44), p=0.044 |
| **FIGO stage** | 13.018 (4.54–37.31), p<0.001 | 2.51 (0.71–8.88), p=0.152 |
| **ER IHC expression** | 4.563 (1.80–11.60), p=0.001 | 1.61 (0.50–5.17), p=0.422 |
| **ER pathway activity** | 5.545 (2.10–14.62), p=0.001 | 2.21 (0.65–7.60). p=0.207 |

All prognostic factors that were significantly associated with disease free and disease specific survival in univariable analysis were included in the multivariable Cox regression analysis. Data from the clinical cohort. Tumor grade: low grade is grade 1 and 2; high grade is grade 3. LVSI: lymphovascular space invasion. Deep MI: Myometrial invasion of >50% of the myometrium. FIGO stage: stage I vs. stage II–IV. ER IHC expression: low immunohistochemical expression of ER (≤10%) vs. high ER IHC expression (>10%). ER pathway activity: low (first quartile of ER pathway activity scores) vs. high (scores in 2^nd^ to 4^th^ quartiles).
